# Wedged Sensor in Distress? Lessons Learned from Troubleshooting Dampened Transmitted PA Waveforms of CardioMEMS Device

**DOI:** 10.1155/2020/3856940

**Published:** 2020-02-11

**Authors:** Robby Singh, Santo Scarfone, Marcel Zughaib

**Affiliations:** ^1^Providence Hospital, Michigan State University, USA; ^2^Georgetown University, USA

## Abstract

*Introduction*. Cardiovascular disease is a leading cause of morbidity and mortality with heart failure constituting a large portion of this spectrum. Heart failure patients have 90-day readmission rates of nearly 41% associated with a high expense. Numerous strategies to reduce readmissions have been attempted with the CardioMEMS pulmonary artery pressure monitoring system as one of the more successful ones. As this device becomes used more frequently, it is important to recognize procedural complications. We present of a rare complication where a patient underwent successful device placement and was subsequently found to have dampened waveforms which were due to device migration. *Case Report*. A 79-year-old male underwent successful CardioMEMS placement due to recurrent heart failure hospitalizations. 6 months later, the transmitted waveforms appeared dampened, and repeat angiography revealed a device that had migrated. Rather than abandoning the device, it was recalibrated and continued to transmit data and helped manage the patient's heart failure. *Conclusion*. CardioMEMS is a cost-effective tool to help reduce heart failure hospitalizations. Device migration is a rare complication and can lead to inaccurate data. However, as seen in this case, the device can be successfully recalibrated and can continue to be utilized to help reduce heart failure admissions.

## 1. Introduction

Cardiovascular disease (CVD) is a leading cause of morbidity and mortality worldwide and in the US and encompasses a wide variety of diseases. Heart failure constitutes a large portion of this spectrum with close to 6 million adults affected in the US [1]. That number is expected to increase over the next 15 years with CVD-related costs projected to reach up to $1.1 trillion, a large portion of which can be attributed to heart failure (HF) rehospitalizations [2]. Clinical trials have shown that HF patients have 90-day readmission rates of nearly 41% with a mean per-patient expense of $16,000 [2]. Further, the average lifetime expense of each HF patient is roughly $83,980, 80% of which is thought to be accumulated during these hospitalizations [2]. During the final two years of life of HF patients, costs are estimated to be $156,000 [3]. Chronic HF cases are expected to reach 8 million by 2030 and HF management costs to increase two- to threefold [3]; patients and healthcare providers alike face a significant financial burden that has been attempted to be addressed by multiple novel strategies.

While guideline-directed medical therapy with regular outpatient follow-up has long been a cornerstone of heart failure treatment, the CHAMPION trial found a significant reduction in heart failure hospitalizations in patients who underwent a CardioMEMS device placement [4, 5]. This pulmonary artery pressure (PAP) remote monitoring device has proven to be an effective tool for treating HF. The PAP sensor system is comprised of a “… coil and a pressure-sensitive capacitor encased in a hermetically sealed capsule covered by silicone” [6]. The CardioMEMS device is placed in the distal pulmonary artery (PA) branch, and the coil and capacitor create an electrical circuit which monitors the pressure fluctuation in the PA as frequencies in the form of wavelengths [6]. This information is paramount in treating heart failure patients with physicians able to make dynamic adjustments of treatment regimens and being able to see nearly real-time changes in patients which help reduce heart failure-related hospitalizations.

Although the CardioMEMS system is a relatively safe device, it can rarely be associated with adverse events, with less than 3% of patients experiencing an adverse event [7]. Pulmonary injury or hemoptysis is the most common complication, roughly 18% of all complications but overall procedure complication incidence of only 2% [6]. Since FDA approval in 2014, thousands of CardioMEMS devices have been implanted worldwide, and there have been only 46 instances of sensor failure, migration, or malfunction, “… of which 35 required recalibrations, 13 reimplantations, and 11 hospitalizations” [7]. We herein present such a case where a patient underwent CardioMEMS device placement and was subsequently found to have dampened waveforms which were found to be a result of device migration.

## 2. Case Report

**Figure 1:**
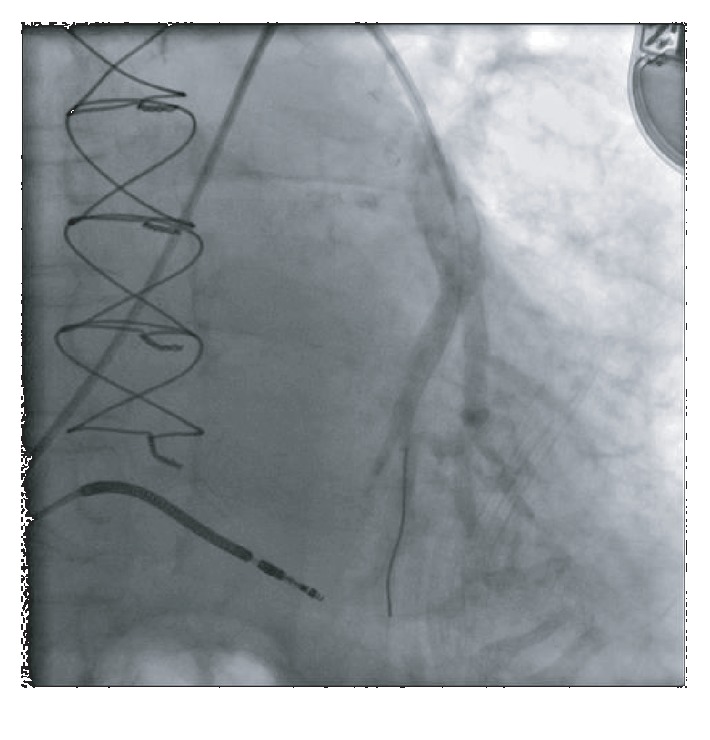
Left pulmonary artery vasculature on angiography revealed suitable vessels for CardioMEMS device placement.

**Figure 2:**
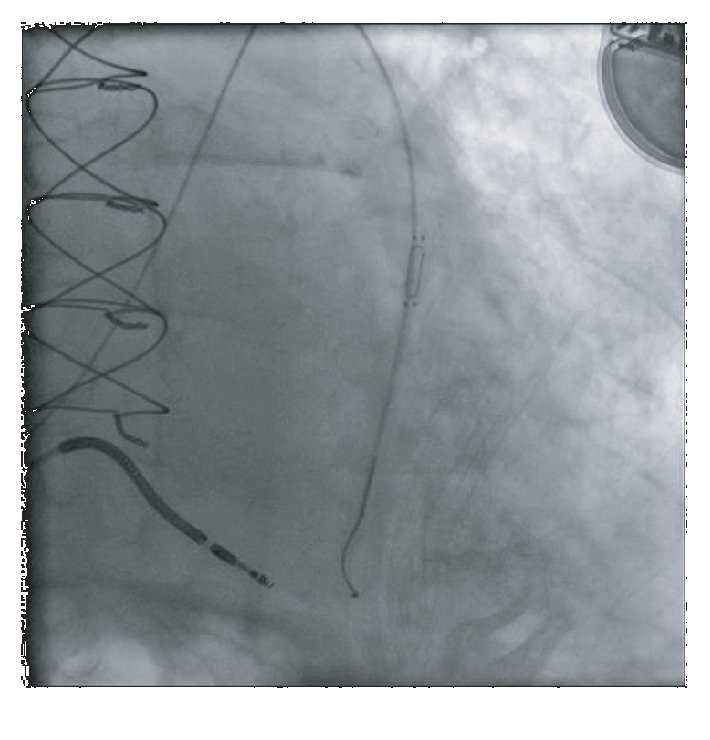
CardioMEMS device successfully deployed.

**Figure 3:**
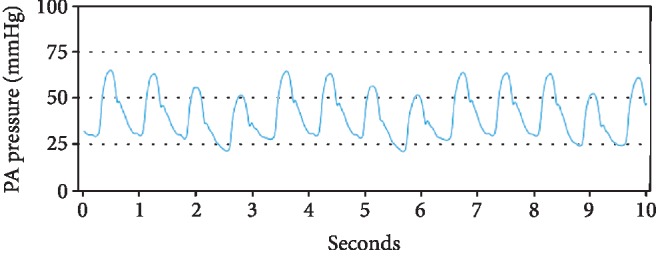
CardioMEMS transmission on the day of implantation showing a good quality waveform.

A 79-year-old male with past medical history of coronary artery disease and prior coronary artery bypass graft surgery, multiple myeloma on long-term immunotherapy, hypertension, diabetes, and cardiomyopathy presented with worsening dyspnea on exertion, peripheral edema, and scrotal edema. The patient had had a heart failure-related hospitalization in the previous year and the presenting NYHA II-III symptoms prompted CardioMEMS device placement. A right heart catheterization with angiography was performed on July 2018 that revealed suitable target vessels for device placement (Figure 1). A CardioMEMS device was successfully deployed (Figure 2), and good quality waveforms were transmitted and recorded (Figure 3). The patient tolerated the procedure well and was discharged safely without complications. For the next 6 months, the patient did not have any heart failure hospitalizations and transmitted daily readings from the CardioMEMS device that revealed a well-controlled PA diastolic pressure.

**Figure 4:**
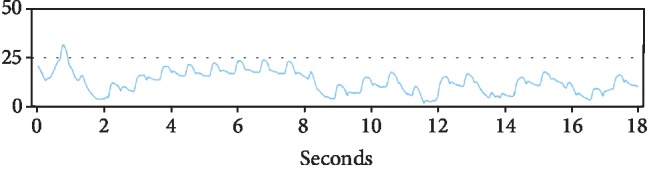
CardioMEMS transmissions 6 months postprocedure showed a dampened waveform compared to baseline.

**Figure 5:**
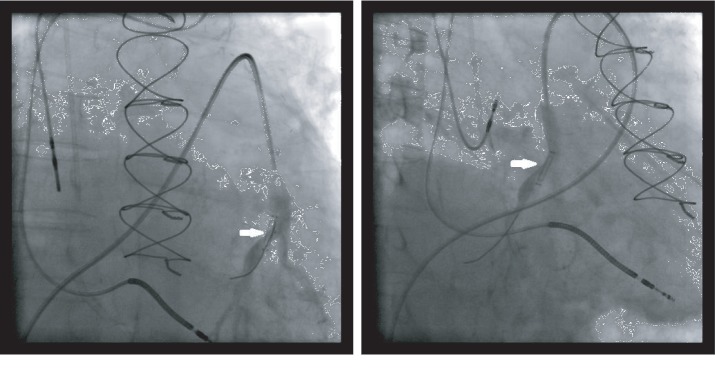
Repeat angiography revealed a migrated CardioMEMS device (arrow) compared to baseline.

**Figure 6:**
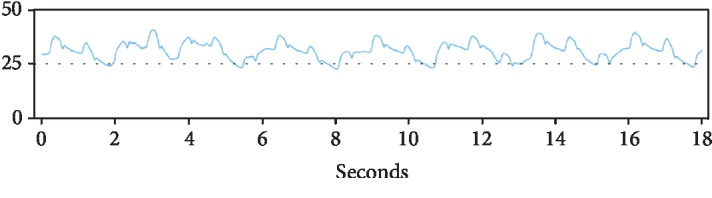
CardioMEMS continued transmission 6 months after recalibration and helped guide heart failure treatment and prevented rehospitalization.

However, 6 months later, the patient's CardioMEMS transmissions were noted to have a dampened waveform (Figure 4). While the patient remained asymptomatic, a possible thrombus was hypothesized as the etiology of the dampening, leading to decreased blood flow in the vessel. The decision was made reevaluate the device by right heart catheterization. Angiography revealed a device that had migrated distally compared to the original implantation site and wedged at the vessel bifurcation (Figure 5). No evidence of thrombus was seen. The device was then recalibrated using the pulmonary artery catheter, but because of the dampening of the waveform, only the PA mean could be recalibrated. The patient was then discharged in stable condition and continued to follow up as an outpatient. 6 months after the recalibration and 1 year after device implantation, the CardioMEMS device continued to transmit data (Figure 6) and the mean PA values helped manage the patient's heart failure and help reduce the risk of repeat heart failure hospitalizations.

## 3. Discussion

Heart failure remains a common cause of cardiovascular morbidity and mortality that places a tremendous burden on healthcare expenditure. The traditional approaches of close outpatient follow-up and guideline-directed medical therapy remain cornerstones of heart failure treatments, but newer technologies have allowed for more dynamic monitoring and, as a result, treatment of the disease. Chief among these, the CardioMEMS remote pulmonary artery pressure monitoring device has been shown to reduce heart failure hospitalizations by 37% in the CHAMPION trial [4, 5]. The recent CardioMEMS postapproval study showed an even greater decrease of 58% in heart failure hospitalizations at 1-year postimplantation [8] while a recent single-center study showed nearly an 80% reduction in heart failure admission and an almost 70% reduction in overall admissions [9].

Consequently, CardioMEMS has become an economically efficient technology, with the cost of device implantation being under $20,000 [1]. In contrast, using the 5-year observational period following the CHAMPION trial, heart failure treatment costs total nearly $170,000 which include future hospitalizations, monthly monitoring, and routine care and “… all costs are weighted based on the assumption that 75% of patients are covered by Medicare and 25% have commercial coverage” [1]. As the technology becomes more widespread, it is important for providers to be aware of the adverse events associated with the device.

Although pulmonary artery injury is the most common complication of the procedure, sensor malfunction is also a possibility, albeit very rare. A thrombus can lead to decreased blood flow and subsequently a dampened waveform. In our patient, we found that the device had likely embolized and wedged at a vessel bifurcation. To help avoid migration of the sensor, it is recommended to implant the device in a vessel with a diameter > 7 mm. However, despite implanting the device in such a location, as was done in our case, it may still not be enough to prevent migration. Using data from a right cardiac catheterization, the device was able to be calibrated and remained a viable tool to manage the patient's heart failure treatment. Conversely, it is also possible to recalibrate the device using noninvasive methods. With echocardiography, a pulmonary regurgitation jet can be used to obtain a mean PA pressure and diastolic PA pressure, which can then be used to calculate a PA systolic pressure. A tricuspid regurgitation jet can also be used to calculate the right ventricle systolic pressure which can substitute for a PA systolic pressure if there is no pulmonic stenosis. All this data can then be used to recalibrate the device, though this less-invasive approach is limited by the ability to acquire good echocardiographic windows. In addition, another possible but unlikely etiology of the dampened waveform may have been partial endothelialization of the device, and the resulting tissue growing over it may have triggered a drift-like process which resulted in inaccurate data. This was supported by cineangiography which showed that the proximal end of the device was free and the distal end was “jammed” in the vessel.

Interestingly, on detailed review of the waveforms after device implantation, a gradual change was noted instead of a sudden dampening. While sensor drift occurs with nearly all implanted sensors, the CardioMEMS device is very stable and only has a reported drift of.1 mm per year [10]. Therefore, the dampened pressures may have been misinterpreted as a well-controlled volume status of the patient and not a device migration. While the PA diastolic pressure is typically used to guide heart failure therapy, monitoring the PA systolic pressure may have alerted the physicians to the gradual decline of the pressure. Once a dampened waveform was identified, it is important to confirm the readings with a right cardiac catheterization. Though in some cases the data may not be useable, there is still an opportunity to recalibrate and utilize the device instead of implanting a new one.

## 4. Conclusion

CardioMEMS remote pulmonary artery pressure monitoring is a cost-effective tool to help reduce heart failure hospitalizations. As the technology gains more widespread acceptance and usage, physicians must be aware of possible adverse events associated with the device. Sensor malfunction is a possible issue that can lead to inaccurate data. We presented such a case where the CardioMEMS device developed a dampened waveform due to device migration and/or partial endothelialization. Despite this, the device can be recalibrated with a right cardiac catheterization and, as seen in our patient, can remain a suitable tool in monitoring and treating heart failure.
